# Early Growth and Neurologic Outcomes of Infants with Probable Congenital Zika Virus Syndrome

**DOI:** 10.3201/eid2211.160956

**Published:** 2016-11

**Authors:** Antonio Augusto Moura da Silva, Jucelia Sousa Santos Ganz, Patricia da Silva Sousa, Maria Juliana Rodvalho Doriqui, Marizelia Rodrigues Costa Ribeiro, Maria dos Remédios Freitas Carvalho Branco, Rejane Christine de Sousa Queiroz, Maria de Jesus Torres Pacheco, Flavia Regina Vieira da Costa, Francelena de Sousa Silva, Vanda Maria Ferreira Simões, Marcos Antonio Barbosa Pacheco, Fernando Lamy-Filho, Zeni Carvalho Lamy, Maria Teresa Seabra Soares de Britto e Alves

**Affiliations:** Federal University of Maranhão, Sao Luis, Maranhão, Brazil (A.A.M. Silva, M.R.C. Ribeiro, M.R.F.C. Branco, R.C.S. Queiroz, M.J.T. Pacheco, V.M.F. Simões, F. Lamy-Filho, Z.C. Lamy, M.T.S.S.B. Alves);; State Department of Health of Maranhão, Sao Luis, Maranhão (J.S.S. Ganz, P.S. Sousa, M.J.R. Doriqui, F.R.V. da Costa, F.S. Silva, M.A.B. Pacheco)

**Keywords:** Zika virus infection, growth, birthweight, epilepsy, microcephaly, congenital abnormalities, neurologic, outcomes, infants, viruses

## Abstract

We report the early growth and neurologic findings of 48 infants in Brazil diagnosed with probable congenital Zika virus syndrome and followed to age 1–8 months. Most of these infants had microcephaly (86.7%) and craniofacial disproportion (95.8%). The clinical pattern included poor head growth with increasingly negative *z*-scores, pyramidal/extrapyramidal symptoms, and epilepsy.

The first reports of Zika virus infection in Brazil were in early 2015 (*1*). Shortly thereafter, Zika virus was associated with microcephaly (*2*). In February 2016, the World Health Organization (WHO) declared the potential association between Zika virus and microcephaly, a public health emergency of international concern (*3*).

Zika virus is able to cross the placental barrier. A growing body of evidence suggests that Zika virus causes cell death in neurons in vitro (*4*), brain anomalies, and microcephaly, resulting in what has been called congenital Zika virus syndrome (*5*). Cortical and subcortical atrophy, brain calcifications, ventriculomegaly, cerebellum anomalies, and abnormal neuronal migration have been described (*6*). The main reported signs and symptoms include abnormalities in neurologic examination, dysphagia, microcephaly (*7*–*9*), and a phenotype characterized as fetal brain disruption sequence (*10*).

Because this congenital infection is newly recognized, its full spectrum is not completely described, and little is known about the growth and neurologic outcomes of infants with congenital Zika virus syndrome in the first months of life. We reviewed the records of 48 infants born from September 2015 onwards that were enrolled at the Reference Center for Neurodevelopment, Assistance, and Rehabilitation of Children during January–May 2016 in Sao Luis, Brazil. 

## The Study

Because isolating Zika virus from human tissues is difficult, we used the following definition by Franca et al. (*6*), which was developed based on a protocol of the Brazil Ministry of Health (*11*) to identify highly probable cases of congenital Zika virus syndrome: 1) central nervous system abnormalities detected by cranial computed tomography (CT) scan, with or without microcephaly; and 2) negative results for syphilis, toxoplasmosis, rubella, cytomegalovirus, and herpes (STORCH) on serologic tests of the infant after delivery. Microcephaly was defined as head circumference (HC) 2 SD below the mean for gestational age and sex based on the INTERGROWTH-21st standards (*12*). Severe microcephaly was defined as HC 3 SD below the mean (*12*). The mothers were asked about the month of appearance of rashes during pregnancy. Birthweight and birth length *z*-scores were also classified according to the INTERGROWTH-21st criterion (*12*). The weight, length and HC after birth were classified according to the WHO standards (*13*). The initial status and rate of change of weight, length, and HC were estimated in a random-intercept multilevel linear regression model by using age in months as an explanatory variable. The Research Ethics Board of the Federal University of Maranhão approved the study (1510305).

**Figure 1:**
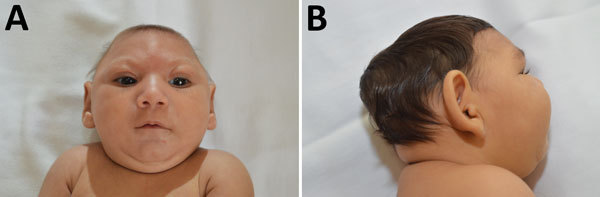
Characteristic phenotype of fetal brain disruption sequence in infants with probable congenital Zika virus syndrome, Sao Luís, Brazil, 2015–2016. A) Craniofacial disproportion and biparietal depression. B) Prominent occiput.

Rash during pregnancy was reported by 73.9% (34/46) of mothers, mostly in the first trimester (52.2%). Most infants (52.1%) were male, and 87.2% were born at term. The HC *z*-score at birth was considered normal for 13.3% of the infants, whereas for 22.2% of the infants, the HC was >2 but <3 SD below the mean. However, most infants had an HC >3 SD below the mean (64.5%). The birth length *z*-score was compromised for 43.2%, and the birthweight was >2 SD below the mean for 19.6% of infants. The mean age at last visit to the reference center was 4.4 months. Nearly all infants had a phenotype characteristic of fetal brain disruption sequence (Figure 1), including craniofacial disproportion (95.8%), biparietal depression (83.3%), prominent occiput (75.0%), and excess nuchal skin (47.9%) (Table).

**Table:** Clinical characteristics of probable congenital Zika virus syndrome in infants from birth to 1–8 months of age, Sao Luis, Brazil, 2015–2016

Characteristic	No. (%)
Rash in mother during pregnancy, n = 46	
First trimester	24 (52.2)
First month	1 (2.2)
Second month	12 (26.1)
Third month	11 (23.9)
Second trimester	10 (21.7)
Fourth month	9 (19.6)
Sixth month	1 (2.2)
No rash	12 (26.1)
Sex, n = 48	
M	25 (52.1)
F	23 (47.9)
Gestational age at birth, n = 47	
Preterm	4 (8.5)
Term	41 (87.2)
Postterm	2 (4.3)
Head circumference *z*-score at birth,* n = 45	
>–2	6 (13.3)
Microcephaly, <–2	10 (22.2)
Severe microcephaly, <–3	29 (64.5)
Birth length *z*-score,* n = 3	
>–2	21 (56.8)
<–2	11 (29.7)
<–3	5 (13.5)
Birthweight *z*-score,* n = 46	
>–2	37 (80.4)
<–2	8 (17.4)
<–3	1 (2.2)
Age at last visit, mo, n = 48	
1	2 (4.2)
2	6 (12.5)
3	7 (14.6)
4	10 (20.8)
5	10 (20.8)
6	7 (14.6)
7	5 (10.4)
8	1 (2.1)
Phenotype, n = 48	
Craniofacial disproportion	46 (95.8)
Biparietal depression	40 (83.3)
Prominent occiput	36 (75.0)
Excess nuchal skin	23 (47.9)
Signs and symptoms, n = 48	
Irritability	41 (85.4)
Pyramidal/extrapyramidal syndrome	27 (56.3)
Epileptic seizures	24 (50.0)
Dysphagia	7 (14.6)
Congenital clubfoot	5 (10.4)
Arthrogryposis	5 (10.4)
Cleft lip/cleft palate	1 (2.1)
Electroencephalogram findings, n = 27	
Abnormal activity, no epileptiform discharges	13 (48.1)
Focal epileptiform discharges	8 (29.6)
Multifocal epileptiform discharges	6 (22.2)
Cranial computed tomography imaging findings, n = 48	
Calcifications in the brain parenchyma	44 (91.7)
Malformation of cortical development	42 (87.5)
Ventriculomegaly	37 (77.1)
White matter attenuation	15 (31.3)
Brain stem and cerebellum hypoplasia	6 (12.5)

*Reported as deviations of the raw *z*-score from the mean measured in SD units.

Of the 48 infants, 85.4% had irritability, making irritability the most common symptom described, followed by pyramidal/extrapyramidal syndrome (56.3%), epileptic seizures (50.0%), and dysphagia (14.6%). Pyramidal syndrome included hypertonia, clonus, hyperreflexia, and increased archaic reflexes. Extrapyramidal symptoms were characterized by tonus fluctuation and asymmetric dyskinesias in the extremities that were absent during sleep. Some infants also had clubfoot (10.4%) and arthrogryposis (10.4%), and 1 infant (2.1%) had cleft lip/cleft palate. Among the 27 infants who underwent electroencephalography, 48.1% had abnormal brain activity without epileptiform discharges, 29.6% had focal discharges, and 22.2% had multifocal epileptiform discharges. All infants had abnormal cranial CT scan imaging findings. The most common were brain calcifications (91.7%), cortical malformations (87.5%), and secondary ventriculomegaly (77.1%). Brain stem and cerebellum hypoplasia and white matter attenuation were less common (Table).

**Figure 2:**
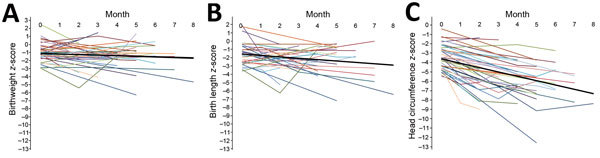
Weight (A), length (B), and head circumference (C) *z*-scores from birth to 1–8 months of age among infants with probable congenital Zika virus syndrome, Sao Luís, Brazil, 2015–2016. The thick black line depicts the mean *z*-score at birth and the mean rate of change in the *z*-score over time, estimated in a random-intercept multilevel linear regression model.

For each infant, we noted weight, length, and HC *z*-scores at birth and each postnatal visit up to 8 months of age (Figure 2). The mean HC *z*-score at birth was −3.61, and it decreased −0.46 per month. The mean weight *z*-score was −1.12 at birth, and it decreased −0.08 per month. The mean length *z*-score was −1.57 at birth, and it decreased −0.16 per month.

## Conclusions

We describe the early growth and neurologic outcomes of infants with probable congenital Zika virus syndrome in the first 8 months of age. In total, 64.5% of infants were born with severe microcephaly, and 95.8% had a phenotype of fetal brain disruption sequence.

The most common clinical symptom noted was irritability, characterized by hyperexcitability (clonus following external stimulation), irritable and impatient cry, and sleep disorders. The infants were difficult to calm down even when fed. As the infants aged, neurologic symptoms began to emerge, usually from the second to the third month onwards with pyramidal/extrapyramidal syndrome, epileptic seizures, and dysphagia, although some infants had >1 of these symptoms much earlier. All infants who underwent electroencephalography had some abnormality, including brain activity maturation disorders and focal or multifocal epileptiform discharges. In 9 infants, brain activity maturation disorders evolved into focal or multifocal epileptiform discharge patterns over time. Focal or multifocal patterns were associated with epileptic seizures that did not respond to medication. Five infants initially had hypsarrhythmia, indicating highly disorganized brain activity, and had spasms and neuromotor delays. These 5 infants subsequently had a multifocal epileptiform pattern.

Early head growth was severely compromised, suggesting a very disruptive brain insult (*10*). In addition, as the infants aged, the HC *z*-scores dropped even further, suggesting that most of these infants would not be able to show catch-up growth. The HC *z*-score was substantially compromised (−5.45) at 4 months of age, whereas the weight *z*-score was in the normal range (−1.44), and the length *z*-score was affected (−2.21) but not as substantially.

Notably, 6 infants with probable congenital Zika virus syndrome who had abnormal imaging findings and a characteristic phenotype were not born with microcephaly. However, 3 infants had microcephaly postnatally. This finding suggests that microcephaly at birth is only 1 of the manifestations of this syndrome (*5*). Therefore, screening should be based not only on HC measurement at birth but also on the phenotype associated with fetal brain disruption sequence and cranial CT scan imaging findings.

Our findings are subject to a few limitations. For some infants, data were missing for some variables. A higher likelihood of selection bias exists because infants with more severe cases tended to be referred to the rehabilitation center. Zika virus infection was not confirmed in any mother, and only 1 infant was IgM positive. Because specific laboratory tests were still ongoing, the case definition might have included patients without Zika virus infection. However, we ruled out the 5 most common causes of congenital infection. Chikungunya incidence was low in the area in 2015 (1.3 cases/100.000) (*14*), and congenital infection caused by this pathogen occurs almost exclusively peripartum and is associated with maternal viremia (*15*). No mother in our case series reported fever or arthralgia near delivery.
